# NGS-based transcriptome profiling reveals biomarkers for companion diagnostics of the TGF-*β* receptor blocker galunisertib in HCC

**DOI:** 10.1038/cddis.2017.44

**Published:** 2017-02-23

**Authors:** Yuan Cao, Rahul Agarwal, Francesco Dituri, Luigi Lupo, Paolo Trerotoli, Serena Mancarella, Peter Winter, Gianluigi Giannelli

**Affiliations:** 1 National Institute of Gastroenterology, IRCCS ‘S. De Bellis', Bari, Italy; 2 GenXPro GmbH, Frankfurt, Germany; 3 Department of Emergency and Organ Transplantation, University of Bari, Bari, Italy; 4 Department of Biomedical Sciences and Human Oncology, University of Bari Medical School, Bari, Italy

## Abstract

Transforming growth factor-beta (TGF-*β*) signaling has gained extensive interest in hepatocellular carcinoma (HCC). The small molecule kinase inhibitor galunisertib, targeting the TGF-*β* receptor I (TGF-*β*RI), blocks HCC progression in preclinical models and shows promising effects in ongoing clinical trials. As the drug is not similarly effective in all patients, this study was aimed at identifying new companion diagnostics biomarkers for patient stratification. Next-generation sequencing-based massive analysis of cDNA ends was used to investigate the transcriptome of an invasive HCC cell line responses to TGF-*β*1 and galunisertib. These identified mRNA were validated in 78 frozen HCC samples and in 26 *ex-vivo* HCC tissues treated in culture with galunisertib. Respective protein levels in patients blood were measured by enzyme-linked immunosorbent assay. SKIL, PMEPA1 ANGPTL4, SNAI1, Il11 and c4orf26 were strongly upregulated by TGF-*β*1 and downregulated by galunisertib in different HCC cell lines. In the 78 HCC samples, only SKIL and PMEPA1 (*P*<0.001) were correlated with endogenous TGF-*β*1. In *ex-vivo* samples, SKIL and PMEPA1 were strongly downregulated (*P*<0.001), and correlated (*P*<0.001) with endogenous TGF-*β*1. SKIL and PMEPA1 mRNA expression in tumor tissues was significantly increased compared with controls and not correlated with protein levels in the blood of paired HCC patients. SKIL and PMEPA1 mRNA levels were positively correlated with TGF-*β*1 mRNA concentrations in HCC tissues and strongly downregulated by galunisertib. The target genes identified here may serve as biomarkers for the stratification of HCC patients undergoing treatment with galunisertib.

Standard therapy for patients with advanced hepatocellular carcinoma (HCC) is currently restricted to the use of sorafenib. However, the drug's severe side effects and the modest improvement of overall survival it provides have signaled an impelling need for better drugs.^1, 2, 3^

In the last decade, transforming growth factor-beta 1 (TGF-*β*1) has been recognized as a key driver in liver fibrosis, resulting in a higher risk of HCC development.^4^^–^^5^,^6^ TGF-*β*1 has a double-sword role in many cancers including HCC: as an onco-suppressor it inhibits cell proliferation, whereas as a tumor promoter it triggers the epithelial–mesenchymal transition, which facilitates tumor spread and metastasis because of a downregulation of E-cadherin.^7, 8^ The mechanisms regulating the switch from onco-suppressor to tumor promoter are still debated. However, in *in vitro* and *in vivo* models two distinct responses to TGF-*β*1 have been demonstrated; the ‘early' response is associated with an epithelial, and the ‘late' response with a mesenchymal, phenotype. These responses are correlated with a better or worse prognosis for HCC patients, respectively.^9^ The small molecule kinase inhibitor galunisertib selectively blocks TGF-*β* receptor 1 (TGF-*β*R1), thereby inhibiting the induction of the canonical Smad-2 signaling pathway. Smad-2 inhibition increases the E-cadherin level, reducing the migratory and invasive capabilities of HCC cells.^10^,^11^ In addition, in different preclinical models galunisertib inhibited HCC progression, decreased tumor growth and metastasis and abolished the cross-talk between the surrounding stroma and the tumor (reviewed in Giannelli *et al.*^12^). Based on these data, galunisertib was tested in a phase 2 clinical trial in patients who failed to react to prior sorafenib treatment (NCT01246986). Preliminary data show promising results in terms of overall patient's survival.^13^ However, no biomarkers are so far available to distinguish those patients that may benefit from the promising drug from those that may not. Therefore, here we aim to discover and validate new biomarkers in preclinical models of HCC for companion diagnostics of galunisertib.

## Results

**Figure 1:**
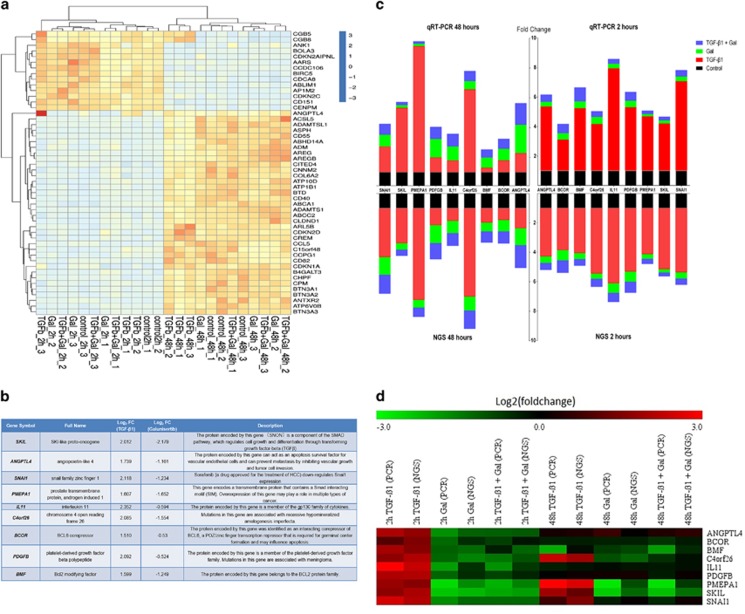
NGS data in HLF cell line from MACE and validation by qRT-PCR. Three replicates were performed for each condition. (**a**) Clustered heat map of normalized sequence counts of the genes with the highest expression as determined by MACE. (**b**) Transcriptional response of selected potential biomarkers to both TGF-*β*1 and galunisertib (gal). (**c**) Comparison of mRNA expression measurement results obtained with either qRT-PCR or MACE. For each gene, the change of expression level is calculated by fold change. (**d**) Expression of mRNA of target genes in comparison with their expression levels in control samples as measured by qRT-PCR. For each gene, the change of expression (−ΔΔCt) is calculated by – (delta Ct target–delta Ct control) and for MACE data the log_2_ fold change is used)

**Figure 2:**
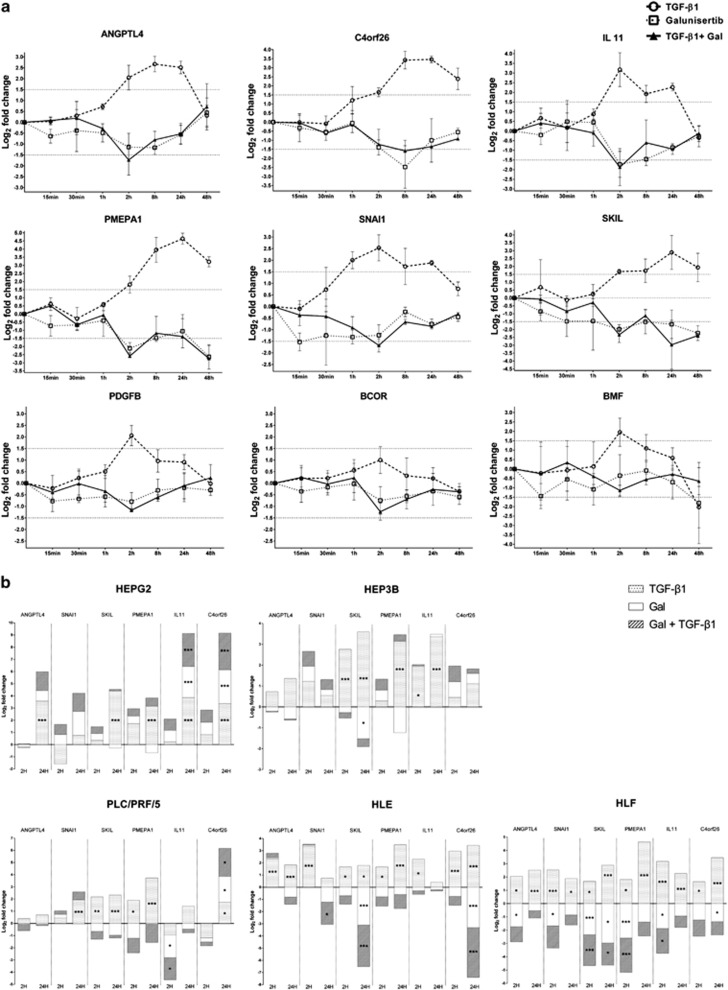
Relative quantitation of mRNA levels of target genes at different time resolutions in HLE, HLF, HepG2, Hep3B PLC/PRF/5 cells. Three replicates were performed for each cell lines. (**a**) Different mRNA levels of target genes in HLF cell line from 15 min to 48 h. For each gene, the change of expression level (−ΔΔCt) for qRT-PCR is calculated by – (delta Ct target–delta Ct control) and the data were shown as mean with S.D. There was no significant change for all the targets within 2 h. Different targets showed different response times to TGF-*β*1 and galunisertib (gal). Some of them even had a significant response after 48 h. (**b**) The mRNA levels of target genes in comparison with the expression levels in control samples in five cell lines after treated for 2 and 24 h. For each gene, the change of expression level (−ΔΔCt) for qRT-PCR is calculated by – (delta Ct target–delta Ct control). Selected targets show a response to TGF-*β*1 and galunisertib both in early TGF-*β* signature and late TGF-*β* signature cell lines. Log_2_ fold change <−1.5 or >1.5 and *P*-value <0.05 (*P<0.05; **P<0.005; ***P<0.0001) were considered significant

As a first step to the discovery of biomarkers for companion diagnostics of galunisertib, genome-wide massive analysis of cDNA ends (MACE) transcription profiles of approximately 152 million mRNAs from the HLF cell line were obtained from three independent samples, each from either untreated controls or cells treated with TGF-*β*1, galunisertib and TGF-*β*1 plus galunisertib after short (2 h) and longer (48 h) incubation, respectively. Hierarchical clustering of the response patterns of the 89 000 most highly expressed transcripts is shown in Figure 1a. As demonstrated, their expression sorts the samples into two clearly distinguished groups corresponding to the 2- and 48-h treatment, respectively. Genes whose log_2_ fold change values were either >1.5 or <−1.5 and *P*-value <0.05 were considered differently expressed. SKIL, Angiopoietin-like 4 (ANGPTL4), SNAI1, PMEPA1, IL11, C4orf26, BCOR, PDGFB and BMF were significantly upregulated in response to TGF-*β*1 and downregulated in response to galunisertib as compared with the control, as shown in Figure 1b. The MACE data were validated by comparison with quantitative real-time PCR (qRT-PCR) expression profiles of the above-mentioned genes. The primers used in each reaction are shown in Supplementary Table 1; data are presented as the log_2_fold change or mean and S.D. The two methods yielded similar results for all candidate biomarkers, as shown in Figures 1c and d. TGF-*β*1 treatment resulted in a significant upregulation of all nine genes within 2 h. However, only PMEPA1, SKIL, C4orf26 and SNAI1 were still upregulated after 48 h. Galunisertib prevented the upregulation of all candidate genes for the 2-h and the 48-h time point both in the presence and the absence of TGF-*β*1. To better resolve the time frame of TGF-*β*1 induction and galunisertib inhibition, respectively, we investigated the abundance of the mRNAs of the candidate genes over a wider time frame ranging from 15 min up to 48 h after treatment in three independent experiments per time point (Figure 2a). Within the first hour after exposure neither TGF-*β*1 nor galunisertib had significant effects, evoking only slight gene expression changes. After exposure to TGF-*β*1 alone, most of the candidate genes displayed the strongest response between 2 and 8 h, and gene expression levels stayed high for up to 24 h. Within this time frame, the addition of galunisertib inhibited the upregulation of these genes. The expression levels of PMEPA1, SKIL, C4orf26 and SNAI1, however, were lowered by galunisertib even after 24 h, but the effect seemed to decrease progressively up to 48 h (Figure 2a). As can also be deduced from Figure 2a, from the selected nine candidate genes BCOR, PDGFB and BMF did not show the expected patterns of robust upregulation by TGF-*β*1 and inhibition by galunisertib in the time resolution experiment and were therefore discarded from further analyses. PMEPA1, SKIL, ANGPTL4, SNAI1, IL11 and C4orf26, however, were considered promising candidates and investigated further.

For this purpose, we measured their expression in four other HCC cell lines: HepG2, Hep3B, PLC/PRF/5 and HLE, which show a TGF-*β*1 response that differs from that of the HLF cell line. In particular, HepG2, Hep3B, PLC/PRF/5 are non-invasive, display an epithelial phenotype and represent the early TGF-*β* signature, whereas HLE and HLF are invasive and represent the late TGF-*β* signature mesenchymal phenotype.^9^ Like the HLF cell line, these cell lines were first treated with TGF-*β*1 and galunisertib and the combination of both, for a shorter (2 h) and a longer (24 h) time. All biomarker candidates responded to TGF-*β*1 and also to galunisertib treatment, but, as expected, there were some differences between invasive and non-invasive HCC cells, according to their early and late TGF-*β* signature.^9^ In the HepG2 and Hep3B cells, for example, the expression of the biomarker candidates after 2 h of TGF-*β*1 treatment was very low, whereas their expression increased after 24 h of incubation. In addition, galunisertib treatment only slightly downregulated the biomarker candidates in HepG2, Hep3B and PLC/PRF/5 cells in comparison with the invasive cell lines. On the contrary, in the invasive HLE cells, the expression of the biomarker candidates was strongly upregulated after both 2 and 24 h of TGF-*β*1 treatment, and strongly downregulated by galunisertib, as in the HLF cells described before (Figure 2b). In conclusion, *in vitro* results show that the expression of our selected biomarker candidates was modulated by galunisertib treatment in both invasive and non-invasive HCC cell lines, but in different time frames and to a different extent.

**Figure 3:**
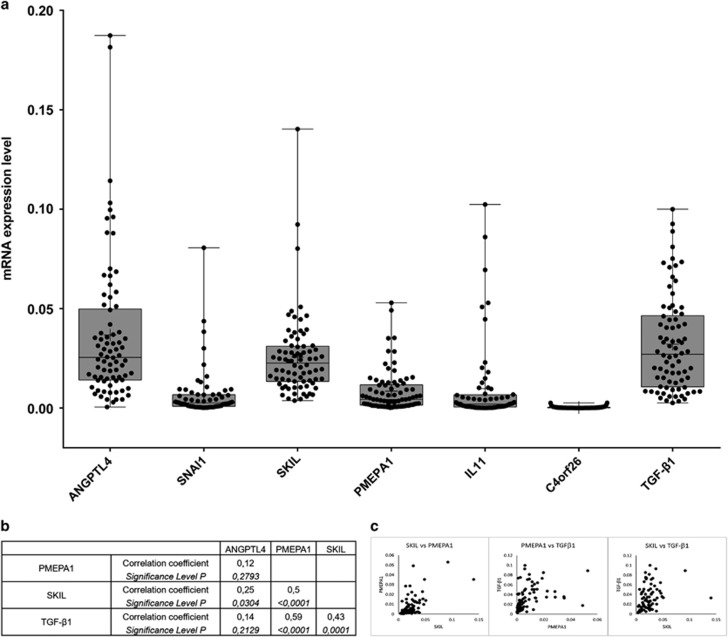
mRNA levels of target genes and analysis of correlation coefficients in HCC tumoral tissues. Three technical replicates were performed for each sample. (**a**) The mRNAs of target genes were measured by qRT-PCR in 78 tissues from HCC patients, the data were shown as mean with range (min to max). ANGPTL4, SKIL and PMEPA1 had high expression levels, whereas SNAI1 and IL11 had a low expression levels. The expression of C4orf26 was undetectable in HCC patients. The expression of TGF-*β*1 was included to analyze the correlation with targets. (**b**: Spearman correlation coefficients to evaluate correlations between the expression levels of the different markers in HCC tissues. (**c**) Scatter plot showing the correlation between PMEPA1/SKIL, PMEPA1/TGF-*β*1 and SKIL/TGF-*β*1 in HCC tumor tissues

To validate the selected biomarker candidates in human liver tissues, we studied their expression in a total of 78 tumoral and peritumoral HCC samples from 55 patients. ANGPTL4, SKIL and PMEPA1 were significantly more strongly expressed than SNAI1, IL11 and C4orf26 (Figure 3a). To better understand such differences, the endogenous TGF-*β*1 levels were measured and correlated with the expression of the potential biomarkers. No significant correlation was found between TGF-*β*1 and ANGPTL4, C4orf26 and IL11 gene expression. The expression of SKIL and PMEPA1, however, was significantly correlated with the TGF-*β*1 concentration in all tumoral tissues (Spearman *r*=0.43, *P*=0.0001; and Spearman *r*=0.59, *P*<0.0001) respectively (Figures 3b and c). Furthermore, a moderate correlation was found between SKIL and PMEPA1 expression (*r*=0.5, *P*<0.0001), whereas the correlation between SKIL and ANGPLT4 mRNA abundance was significant but with a lower coefficient (*r*=0.25, *P*=0.0304). No correlation was found between biomarker expression and histological characteristics of the tissues. As a consequence of their low expression and indemonstrable correlation with TGF-*β*1 levels in human HCC tissues, SNAI1, IL11 and C4orf26 were removed from the list of candidate markers.

**Figure 4:**
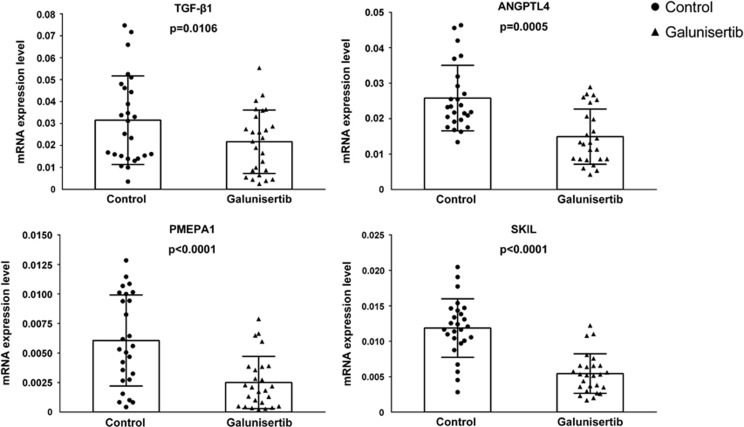
Bar plot for biomarkers of mean value and 95% confidence interval of the mean concentration in the *ex-vivo* model. Three technical replicates were performed for each sample. Relative quantitation of mRNA levels of target genes in resected tissues treated with galunisertib compared with the expression in control samples and the data were shown as mean with S.D. Freshly resected tissues from 26 HCC patients were cultured alive in specific culture medium conditions and treated for 48 h with 10 *μ*M galunisertib. Expression levels of three potential targets, as well as the TGF-*β*1 expression level, show a significant decrease under galunisertib treatment

**Figure 5:**
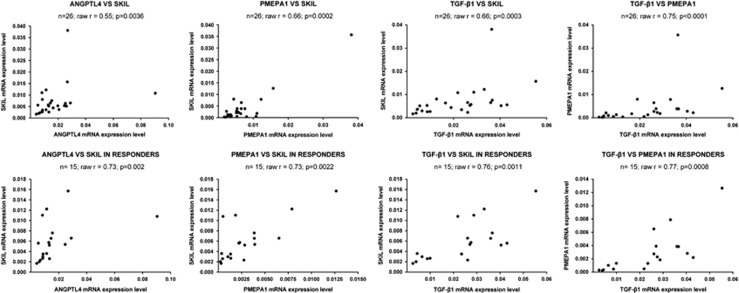
Selected potential biomarkers show higher correlation coefficients in *ex-vivo* galunisertib treated tissues. Three technical replicates were performed for each sample. Pearson correlation coefficients describing the correlations among all biomarkers in the *ex-vivo* tissues. Such coefficients were even stronger in the responder group. Scatter plots describing the correlations between the biomarkers ANGPTL4/SKIL, PMEPA1/SKIL, TGF-*β*1/SKIL and TGF-*β*1/PMEPA1 in all *ex-vivo* tissues and in the responder subgroup. The tumor tissues from 15 of the 26 patients tested responded to galunisertib treatment with a decrease of TGF-*β*1 mRNA

To test the hypothesis that the expression of the identified biomarkers is sensitive to galunisertib also in human HCC specimens, we used an *ex-vivo* assay in which resected tumor tissues were cultured in the presence of galunisertib. After 48 h of incubation, tissues were collected for RNA isolation and analysis. Galunisertib significantly inhibited the expression of all the biomarkers and in addition, the drug significantly (*P*=0.0106) reduced the mean concentration of TGF-*β*1. Similarly SKIL, PMEPA1 and ANGPTL4 were also significantly downregulated by the drug (Figure 4). The analysis of correlation coefficients in all 26 tissues of the *ex-vivo* model revealed statistically significant correlations between SKIL and ANGPTL4 (*P*=0.0036), SKIL and PMEPA1 (*P*=0.0002), SKIL and TGF-*β*1 (*P*=0.0003) and PMEPA1 and TGF-*β*1 (*P*<0.0001). However, in the *ex-vivo* models, not all the tumor tissues showed a response to the galunisertib treatment. For instance, only 15 of the 26 patient tissues (60%) responded to treatment with a decrease of the TGF-*β*1 mRNA. In the responder subgroup, correlation coefficients were even more significant: SKIL and ANGPTL4 (*r*=0.73, *P*=0.002), SKIL and PMEPA1 (*r*=0.73, *P*=0.002), SKIL and TGF-*β*1 (*r*=0.76, *P*=0.0011), PMEPA1 and TGF-*β*1 (*r*=0.77, *P*=0.0008) (Figure 5).

**Figure 6:**
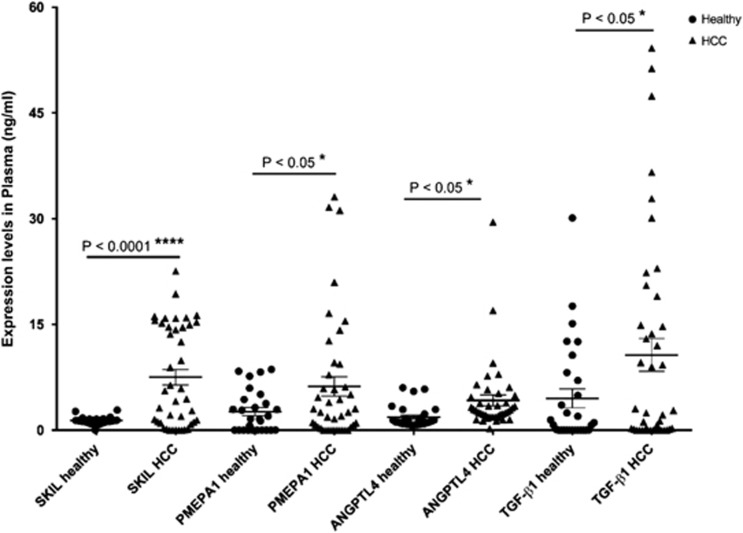
Comparison of the markers expression level at plasma level between the patients with HCC and healthy controls. Three technical replicates were performed for each sample. All three biomarkers had a higher significant average plasma expression levels in patients with HCC compared with healthy controls. *P*<0.0001 and *P*<0.05, respectively

Finally, we measured the concentrations of the proteins encoded by the selected biomarker transcripts in the plasma of 42 HCC patients and 29 healthy controls. In the HCC patients group, SKIL, PMEPA1 and ANGPTL4 plasma levels were significantly higher than in the healthy controls. Similarly, also TGF-*β*1 levels were higher in HCC patients compared with the healthy control group (*P*<0.05) (Figure 6). In short, 19% of the HCC patients had higher plasma levels of all four biomarker proteins, whereas in 50% of patients three biomarker proteins were increased.

Spearman correlation coefficients were observed between mRNA levels in tumor tissues and plasma proteins from HCC patients (Table 1). ANGPTL4 mRNA and plasma protein were even inversely correlated (*r*=−0.43, *P*=0.0282). Moreover, there was no statistically significant correlation between the marker proteins in the plasma (Table 2) as only a low positive Spearman correlation (*r*=0.42, *P*=0.0331) was found between SKIL and ANGPTL4. In conclusion, biomarker mRNA expression but not their plasma protein concentrations are correlated with galunisertib effectiveness.

## Discussion

Personalized treatment of patients with HCC is necessary due to the great heterogeneity of the cancer.^14^ Therefore, some studies have grouped HCC patients according to their gene expression characteristics indicating different prognosis and survival.^15, 16, 17^ However, reports on biomarkers to select patients that will benefit from systemic therapy are scarce. Biomarkers attempting to predict the response of patients to the standard sorafenib treatment failed in clinical trials, nor was c-Kit predictive as a pharmacological target of the drug.^18^ The expression of c-MET, however, was correlated with the response to tivantinib and overall survival,^19^ indicating that generating companion diagnostics biomarkers for drugs for the treatment of HCC is possible in principle.

Galunisertib inhibited tumor progression of both pancreatic and HCC carcinomas in preclinical experimental models^12, 20, 21^ and is currently under investigation in clinical phase I/II trials (NCT01246986, NCT02423343, NCT02178358, NCT02240433; https://clinicaltrials.gov). Although preliminary data are promising,^13^ the development of biomarkers for assessing the drug's efficiency is still at an early stage. Indeed, circulating TGF-*β* and related molecules like E-cadherin are not correlated with BCLC classification or with survival, but rather with the biological characteristics of the tumor.^22^

In this study, we show that the expression of the mRNAs of SKIL, PMEPA1 and ANGPTL4 is downregulated by galunisertib in some but not all HCC tissues. The SKI-like (SKIL) gene is the target gene of the TGF-*β*/SMAD pathway and encodes the Ski-related novel protein N (SnoN) that antagonizes TGF-*β* signaling. Several studies have shown that TGF-*β*1 positively regulates SnoN expression at the transcriptional level and SKIL is negatively regulated by the transcriptional cofactor complex SnoN-SMAD4.^23, 24, 25^ Furthermore, SnoN also activates the p53 pathway and may have an important role in regulating tumorigenesis.^26^

Only one study so far investigated the expression of SKIL in 32 HCC tissues, but the authors did not find any role for this gene.^27^ On the contrary, in our study we significantly expanded the number of patients investigated at mRNA and plasma protein levels and we report a robust positive correlation of SKIL and TGF-*β*1 mRNA expression in HCC tumor samples. The strong difference of SnoN plasma concentrations between HCC patients and healthy controls also suggests a role for the SKIL gene in HCC.

The prostate transmembrane protein, androgen induced 1 (PMEPA1), is another important target gene of TGF-*β* signaling and highly expressed in many types of cancers including colon, breast, lung and prostate cancer,^24, 28, 29, 30^ whereas reports about the expression of PMEPA1 in HCC patients are lacking.

ANGPTL4 has an important role in cancer, especially in tumor metastasis, but little is known about its function in HCC metastasis.^31^ The induction of ANGPTL4 by TGF-*β* via the SMAD signaling pathway activates cancer cells for metastasis to the lungs in breast cancer.^31^ In our study, we found that ANGPTL4 mRNA expression was significantly correlated with TGF-*β*1 mRNA after galunisertib treatment in responders, and in HCC patients, plasma ANGPTL4 protein levels were significant higher than in controls.

In *ex-vivo* models, after galunisertib treatment, about 48% of HCC tissues showed a reduced concentration of TGF-*β*1 mRNA. However, the wide variation in TGF-*β*1 mRNA levels precludes the use of TGF-*β*1 mRNA as the sole biomarker for accurate patient selection. Our data suggest that SKIL and PMEPA1 mRNA concentrations in combination with TGF-*β*1 mRNA could be important biomarkers for selecting patients more likely to respond to treatment with the TGF-*β*RI blocker galunisertib, although the mRNA levels of these biomarkers do not correlate with the levels of circulating proteins in patients. This may be because the protein concentration is not only regulated by transcription but also by many other processes (e.g., post-transcriptional and translational regulation and degradation etc.). In conclusion, this study offers a scientific rationale for personalizing therapy by stratifying patients according to their individual level of activation of the TGF-*β* pathway.

## Materials and methods

### Cell culture and drug treatment

Human HCC cell lines were purchased from ATCC (Manassas, VA, USA) (HepG2, PLC/PLF/5 and Hep3B) or from the JCRB cell bank (HLE and HLF). HLF cells were used for genome-wide transcriptome profiling using MACE, whereas the other cell lines were used for the validation of selected potential biomarkers. Galunisertib was kindly provided by Lilly (Indianapolis, IN, USA). Human recombinant TGF-*β*1 was purchased from Peprotech (Rocky Hill, NJ, USA). Before treatment with TGF-*β*1 or galunisertib, cells were washed with PBS buffer and further cultured for 24 h in serum-free conditions to minimize the influence of fetal bovine serum. HCC cells were then either treated with galunisertib (10 *μ*M) alone, TGF-*β*1 (5 ng/ml) alone or with galunisertib plus TGF-*β*1. Untreated cells served as controls. The cells were exposed to the substances for 15 min, 30 min, 1 h, 2 h, 8 h, 24 h and 48 h before trypsinization and isolation of RNA. Identification and validation of other cell lines (HepG2, Hep3B, PLC/PRF/5 and HLE) were performed using the same procedure as described above. All *in vitro* experiments were performed in three replicates.

### RNA isolation and qRT-PCR

Total RNA was isolated using the NucleoSpin miRNA kit (MACHEREY-NAGEL, Duren, Germany). Reverse transcription (RT) of 1.5 *μ*g total RNA was performed using the High-Capacity cDNA Reverse Transcription Kit (Applied Biosystems, Foster City, CA, USA) with RNase Inhibitor (Applied Biosystems). Quantitative real-time PCR was performed using 25 ng cDNA for each reaction and three technical replicates for each sample. All PCR reactions were carried out using the iTaq Universal SYBR Green Supermix (Bio-Rad, Hercules, CA, USA) according to the manufacturer's instructions. The amplification process was conducted at 95 °C for 1 min, followed by 40 cycles at 95 °C for 1 min, 95 °C for 15 s, 60 °C for 1 min. Glyceraldehyde-3-phosphate dehydrogenase (GAPDH) mRNA was used as housekeeping control.

### Transcriptome profiling using MACE

MACE is a 3'-end targeted tag-based reduced representation transcriptome profiling technique that can reliably quantify all poly-adenylated transcripts including those with low expression. Preparation and next-generation sequencing (NGS) of MACE libraries were performed using the MACE kit (GenXPro GmbH, Frankfurt, Germany) according to the manual provided with the kit and essentially as described.^32^ A total of 152 million single 3'-end reads obtained from 24 mRNA sequencing libraries (total 8 conditions with 3 replicates) was initially filtered to eradicate adaptor sequences. Duplicate reads generated by PCR during library preparation were recognized by the TrueQuant technology included in the kit and also removed from the data set. Filtered reads were aligned to the human reference genome (hg19) using novoalign. Then, we used htseq-count to generate gene-enriched count data from aligned bam files and hg19 refseq annotation GTF files. Those reads that could not be mapped to the reference genome were extracted and processed with a customized ClusterBED pipeline to generate annotation and mapped bam files. Still unmapped reads were further aligned with transcriptome assembly using novoalign to generate individual mapped bam files from which we extracted the remaining unaligned reads. These were *de novo* assembled and aligned using a customized BLAST-based assembly workflow employing trinity, blastx and novoalign. Finally, all bam files of each MACE library were combined individually to generate merged gene-enriched count data. Count data were normalized with the geometric mean method and tested for differential expression using the DEseq package.

### Clinical resected HCC tumor tissues and *ex-vivo* HCC tissue profiling

A total of 78 human HCC samples including tumoral and peritumoral tissues were obtained from the Policlinico Hospital in Bari, Italy, between 2010 and 2016 and stored in liquid nitrogen until use. The HCC tumoral tissue were cultured in serum-free conditions, in the presence of 10 *μ*M galunisertib, renewing the medium and drug every 24 h as previously described.^33^ After 48 h of exposure, RNA was extracted and used to assess the expression of selected potential companion diagnostics markers. Gene expression was normalized *versus* the GAPDH housekeeping gene, and the results were referred to the proper untreated control.

### Enzyme-linked immunosorbent assay (ELISA)

SKIL, PMEPA1, ANGL4 and TGF-*β*1 protein levels were measured using commercially available ELISA kits purchased from MyBioSource (San Diego, CA, USA), and R&D Systems (Minneapolis, MN, USA), according to the supplier's recommendations. Blood samples were collected from 42 HCC patients and 29 healthy volunteers. The samples were centrifuged for 10 min at 1500 r.p.m. at room temperature and plasma was separated and stored at −20 °C until use. All assays were performed according to the manufacturer's instructions. Absorption was read at 450 nm using an ELISA plate reader (Bio-Rad).

### Statistical analysis

All *in vitro* experiments involving gene profiling analysis were based on three independent experiments. In the analysis of tumor tissues from HCC patients, data were not normally distributed, and results are described as median and interquartile range. The correlation between marker gene expression levels was assessed as Spearman correlation coefficient. Comparisons for paired groups were done with the Wilcoxon test for paired data. In *ex-vivo* analyses, Gaussian distributed variables are described as mean and 95% confidence interval, whereas non-Gaussian distributed variables were transformed into natural logarithms and described as geometric mean and 95% confidence interval. To evaluate differences between control and treated patient tissues, *t*-test for paired data were used. Correlations among variables were evaluated by Pearson correlation coefficients. Statistical analyses of blood samples from HCC patients were expressed as mean±S.E.M. All analyses were performed using SAS 9.4 for Windows (Cary, NC, USA)/GraphPad Prism 6 (San Diego, CA, USA). *P*-values<0.05 were considered statistically significant.

## Figures and Tables

**Table 1:** Spearman correlation coefficients to evaluate correlations between potential targets at tissue and plasma levels

	**ELISA ANGPTL4**	**ELISA PMEPA1**	**ELISA SKIL**	**ELISA TGF-*****β*****1**
*mRNA ANGPTL4*				
Correlation coefficient	−0.43	0.09	−0.11	−0.27
Significance level *P*	0.0282	0.6446	0.591	0.1781
				
*mRNA PMEPA1*				
Correlation coefficient	0.20	0.38	0.24	−0.12
Significance level *P*	0.3189	0.0589	0.2366	0.5661
				
*mRNA SKIL*				
Correlation coefficient	−0.02	0.06	0.11	0.24
Significance level *P*	0.9195	0.772	0.591	0.2328
				
*mRNA TGF-*β*1*				
Correlation coefficient	0.06	0.11	0.39	0.02
Significance level *P*	0.7639	0.5923	0.0504	0.9326

**Table 2:** Spearman correlation coefficients to evaluate the correlations between potential targets at plasma levels

	**ELISA ANGPTL4**	**ELISA PMEPA1**	**ELISA SKIL**
*ELISA PMEPA1*			
Correlation coefficient	−0.05		
Significance level*P*	0.7926		
			
*ELISA SKIL*			
Correlation coefficient	0.42	0.24	
Significance level *P*	0.0331	0.2473	
			
*ELISA TGF-*β*1*			
Correlation coefficient	−0.09	−0.1	0.27
Significance level *P*	0.6406	0.6278	0.1847
