# Supplementary material for: NGS-based transcriptome profiling reveals biomarkers for companion diagnostics of the TGF-β receptor blocker galunisertib in HCC
Source: Cell Death Dis. 2017 Feb 23;8(2):e2634–. doi: 10.1038/cddis.2017.44 (PMC5386488; doi:10.1038/cddis.2017.44)
Supplement: Supplementary Table 1 [file cddis201744x1.docx]

**Table 1 Supplementary.** Target genes and their primer sequence for qRT-PCR

| Gene symbol | Primer Sequence (5' -........-3') |
| --- | --- |
| ANGPTL4 | Fwd: CCACGAAAGACGGTGACTCT  Rev: CACTCAGAAAGGGGGCTTC |
| BCOR | Fwd: CTGACCCCACCTTGGCTAC  Rev: CATCATTGCGACCCTGGA |
| BMF | Fwd: TGGTGGCAGATCCTCCTCTT  Rev: TTCCAGACGGTGTTCCTGGT |
| C4orf26 | Fwd: GTTGGTGGTAACTGTGGCAGAAG  Rev: GGTGGAGGGGTGAGTGTAAAGA |
| IL11 | Fwd: TCATTCAGGGAGGCTAAGGA  Rev: CCCAAAGTGCCAGGATTACA |
| PDGFB | Fwd: TCCTTGTGGACTGGCTGTG  Rev: GGACTTTGGGAAATGGAGGT |
| PMEPA1 | Fwd: AGTGTTGAAGCCCAGTGTCC  Rev: CAGCAGAGAAGCCGAGAG |
| SKIL | Fwd: ATTTCCACCAGTTCTCTTCTTTTT  Rev: ACAACACATTCTTCTTCCCCACT |
| SNAI1 | Fwd: GCTATTTCAGCCTCCTGTTTG  Rev: AGACCAGAGCACCCCACTT |
